# Cardiovascular magnetic resonance measures of coronary artery blood flow improve after receipt of oral conjugated estrogen and/or high dose atorvastatin in early post-menopausal women without known coronary arteriosclerosis

**DOI:** 10.1186/1532-429X-11-S1-P160

**Published:** 2009-01-28

**Authors:** Chirapa Puntawangkoon, Tim M Morgan, David M Herrington, Craig A Hamilton, William G Hundley

**Affiliations:** Wakeforest University, Winston Salem, NC USA

**Keywords:** Atorvastatin, Cardiovascular Magnetic Resonance, Cardiac Magnetic Resonance, Conjugate Equine Estrogen, Early Postmenopausal Woman

## Objectives

To determine the effect of oral hormone replacement with conjugated equine estrogens (CEE, 0.625 mg po qd) and/or high dose atorvastatin (ATORV, 80 mg po qd) on submaximal exercise induced coronary artery blood flow (CABF) in relatively early postmenopausal women without documented coronary artery disease (CAD).

## Background

Oral conjugated equine estrogens and HMG-CoA reductase inhibitor (statin) therapy have been shown to enhance arterial endothelial function, but the effect of these agents on submaximal coronary artery blood flow is unknown.

## Methods

We randomized 56 postmenopausal women aged 59 ± 3 years without evidence of coronary arteriosclerosis, into a randomized double blinded, cross-over study in which women were assigned to receive 8 weeks of oral CEE 0.625 mg or placebo, with or without 80 mg per day of ATORV. Each treatment period was separated by a 6 week "washout" period of no treatment. Prior to receipt of any therapy, and after each treatment period, each woman underwent rest/submaximal exercise measures of CABF in the left anterior descending coronary artery using phase contrast cardiac magnetic resonance imaging at 1.5 T. Exercise was performed on an electronically braked bike (Lode, Netherlands). Scan parameters included an 8-mm-thick slice, a 256 × 256 matrix, a 20-cm FOV, a through-plane velocity encoding of 150 cm/s, a 13.8-ms TR, and a 6.7-ms TE.

## Results

Of 56 women, 47 were compliant with therapy (85% of administrated medication) and their results are displayed below (least square mean ± standard error).

## Conclusion

**Figure 1:**
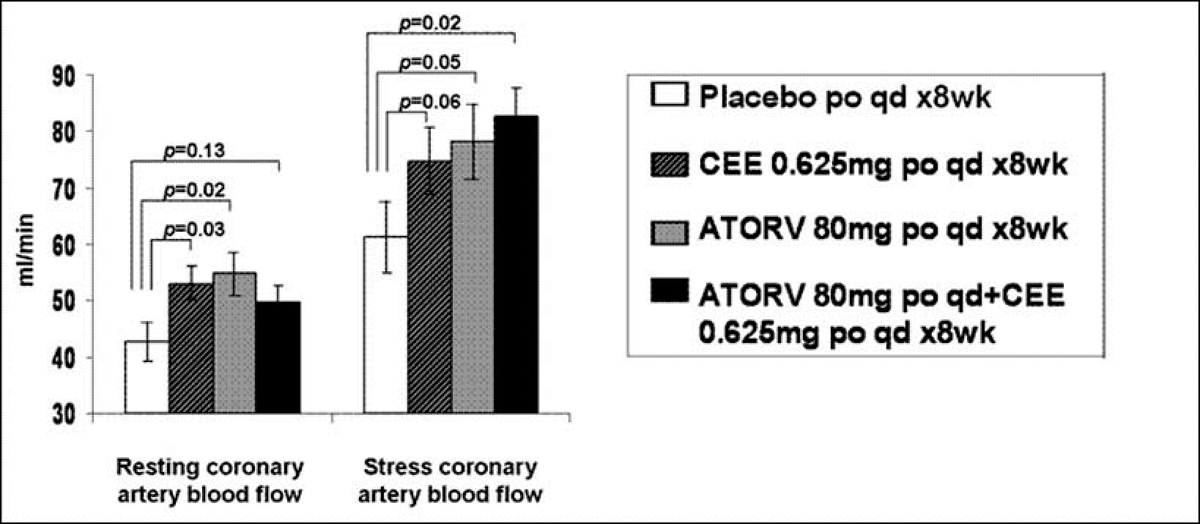
**Left anterior descending coronary artery blood flow (ml/min)**.

In early postmenopausal women without coronary arteriosclerosis, the administration of conjugated equine estrogens 0.625 mg, Atorvastatin 80 mg, or their combination have favorable effects on resting and/or submaximal exercise-induced coronary artery blood flow (Figure 1).

